# Pathogenetic approach to the treatment of functional disorders of the gastrointestinal tract and their intersection: results of the Russian observation retrospective program COMFORT

**DOI:** 10.1186/s12876-019-1143-5

**Published:** 2019-12-31

**Authors:** Vladimir T. Ivashkin, Elena A. Poluektova, Alexey B. Glazunov, Mikhail A. Putilovskiy, Oleg I. Epstein

**Affiliations:** 1 I.M. Sechenov First Moscow State Medical University (Sechenov University), 8–2, Trubetskaya St, 119991 Moscow, Russian Federation; 2 Department of Medical Information, Research and Production Company Materia Medica Holding LLC “NPF” MATERIA MEDICA HOLDING, 9, 3rd Samotyochny Per, Moscow, Russian Federation 127473; 3 The Institute of General Pathology and Pathophysiology, 8, Baltiyskaya St, Moscow, Russian Federation 125315

**Keywords:** Irritable bowel syndrome, Functional dyspepsia, Overlap of irritable bowel syndrome and functional dyspepsia, “7*7” questionnaire

## Abstract

**Background:**

The aim of this study was to investigate the efficacy and safety of the novel complex drug, consisting of released-active form of antibodies to S-100 protein, tumor necrosis factor-α and histamine, (Kolofort) under outpatient conditions in patients with functional dyspepsia (FD), irritable bowel syndrome (IBS), and FD-IBS overlap.

**Methods:**

The subjects of the observational noninterventional retrospective program were the data of 14,362 outpatient records of patients with diagnosed FD, IBS, and/or overlap, who were observed by gastroenterologists from November 01, 2017, through March 30, 2018, who received the drug Kolofort in monotherapy for 12 weeks, 2 tablets twice a day. To assess the presence and severity of symptoms of functional gastrointestinal disorders (FGID), the “7*7” questionnaire developed by a working group from the Russian Gastroenterological Association was used. The evaluated parameters included the proportion of patients: who had a 50% or more reduction in the total score; who have switched to the less severe category of the condition; who have switched to the “healthy” or “borderline ill” severity categories; and the change in the score in domains 1–7.

**Results:**

The final efficacy analysis included data from 9254 patients. A decrease in the total score by 50% or more was observed in 80.45% of patients with FD, 79.02% of patients with IBS, and in 83% of patients with both IBS and FD. Switch to a lower severity category of the condition at the end of therapy was noted in 93.35% of patients with FD, in 93.80% of cases in patients with IBS, and in 96.17% of cases in patients with a combination of IBS and FD. A total of 94 adverse events (AEs) were reported in 80 patients (0.65%).

**Conclusion:**

The COMFORT program has demonstrated the positive effect of treatment in the majority of patients with IBS and FD and their combination in real clinical practice.

## Background

A variety of clinical forms and the heterogeneity of the pathogenetic mechanisms of functional gastrointestinal disorders (FGID) complicate the diagnosis and choice of an effective treatment regimen [1]. Irrational pharmacotherapy, the prescription of symptomatic drugs that do not have indications for treatment of the FGID, leads to polypharmacy, low patient adherence to treatment, and an increased risk of developing adverse events (AE) [2–5].

Particular difficulties in the treatment of FGID arise from the combination of their various forms [3]. The association of IBS FD is most commonly observed [6]. Such patients have elevated visceral hypersensitivity, greater severity of gastrointestinal symptoms, and lower quality of life than patients with a single FGID [7, 8].

In the FD, dysfunction of the digestive tract organs is often combined with a mental illness [9, 10]. According to the literature, up to 90% of patients with FGID have concomitant psychiatric disorders [11, 12]. This neuropsychological component also serves as a key link in the pathogenesis of the combination of FD and IBS [13, 14].

Chronic inflammation in the gastrointestinal tract (GIT) caused by imbalances of pro-inflammatory and anti-inflammatory factors (tumor necrosis factor-α (TNF-α); interleukins (IL) IL-2, IL-6, IL-10, and histamine) plays an important role in the development and progression of FGID [15, 16].

Due to the pathogenetic mechanisms associated with impaired motor function of the GIT and a reduced threshold for the perception of stimuli, abdominal pain appears to be the main symptom of most of FGID [17, 18].

Currently, various symptomatic and disease-modifying approaches are being used for the treatment of FGID: antispasmodics, proton pump inhibitors, drugs that relieve diarrhea/constipation, prokinetics, probiotics, antidepressants, antagonists of 5-HT_3_ and 5-HT_4_ receptors, opioid receptor agonists, and selective activators of C-2 chloride channels [19–21]. Most of these drugs, however, are not always able to effectively solve the problems of patients. In this regard, in the routine practice of gastroenterologists, therapists, and general practitioners, there is a need for a multi-targeted drug affecting the main pathogenesis of FGID.

For the treatment of FGID, the combination of released-active form of antibodies to S-100 protein, TNF-α and histamine (RAF of Abs to S 100, Abs to TNF-α and Abs to H), a pathogenetically targeted drug Kolofort, was developed by the Research and Production Company Materia Medica Holding (LLC NPF” MATERIA MEDICA HOLDING”) Moscow, Russia and introduced into practical medicine. The RAF of Abs in the drug provide an anti-inflammatory, spasmolytic, and anxiolytic effect [22].

It was established experimentally that the antispasmodic effect of the combination of RAF of Abs to S 100, Abs to TNF-α and Abs to H is due to the relaxation of smooth muscles and a decrease in the tone of the walls of the stomach and intestines. Anti-inflammatory properties are realized due to the effect of the drug on the production of TNF-α and it’s associated cytokines. The positive effect of the drug components on the nervous and humoral regulation of functions of the gastrointestinal tract has been confirmed [22].

A randomized, placebo-controlled clinical study has demonstrated the efficacy and safety of combination of RAF of Abs to S 100, Abs to TNF-α and Abs to H for the treatment of FGID [22, 23]. At the same time, there was no large-scale population-based research of the combination of RAF of Abs to S 100, Abs to TNF-α and Abs to H under outpatient conditions in patients with FD, IBS, and their combination.

## Methods

### Study design

This was an observational nonintervention retrospective program to study the efficacy and safety of the use of the combination of RAF of Abs to S 100, Abs to TNF-α and Abs to H in patients with FD, IBS, and their combination in outpatient settings. In order to obtain a representative sample, data of 14,362 patients from 67 cities of the Russian Federation were collected. Four hundred seventy-three gastroenterologists participated in the Russian Observational Program COMFORT.

The study included the analysis of medical records of outpatients 18 and older, of both genders, diagnosed with FD or IBS or with a combination of FD and IBS, as verified by their medical history. To be included in the study, patients had to be observed by a gastroenterologist from November 01, 2017, through March 30, 2018. Patients were examined in accordance with the recommendations of the Russian Gastroenterological Association for the treatment of FD and IBS.

FD is characterized by the presence of permanent or periodic symptoms of dyspepsia, such as upper abdominal pain, nausea, a feeling of burning in the stomach area, fullness in the stomach, early satiety, which appeared at least 6 months before diagnosis, lasting at least 3 months, in the absence of an organic disease that explains the appearance of these symptoms.

Based on the Rome IV Criteria IBS is manifested by a symptom-based scheme requiring that patient complains of abdominal pain on average at least once per week and that pain is associated with two or more of the following characteristics: it is related to defecation; to a change in the frequency of stool; or it is connected with a change in the form of the stool. These criteria should be fulfilled for the last 3 months, with symptom onset at least 6 months before diagnosis.

In addition, previous treatment with other drugs have to be continued and during the observation period patients to be treated with the combination of RAF of Abs to S 100, Abs to TNF-α and Abs to H for 12 weeks, 2 tablets twice a day in accordance with instruction for medical use.

The COMFORT Observation Program has been approved by the Independent Interdisciplinary Committee for the Ethical Review of Clinical Studies of the Russian Federation. The observational type of the study did not imply additional methods of laboratory or instrumental examination for the inclusion of patient’s data in the program. Patients with decompensated or unstable somatic disease, patients showing alarm symptoms, patients with significant accompanying gastrointestinal or other diseases, pregnant or nursing women were not admitted to the study.

To assess the presence and severity of symptoms characteristic of FGID, the “7*7” questionnaire was used. The “7*7” questionnaire was developed by the Russian Gastroenterological Association based on clinical symptoms described in the Rome III criteria and recommended for use by gastroenterologists in routine practice to assess the presence and severity of the seven main symptoms of FGID observed over the past 7 days [24]. The first four domains in the “7*7” questionnaire are considered as symptoms of FD; domains 5 through 7 characterize IBS symptoms.

Patients completed the questionnaire before and 3 months after the therapy with the combination of RAF of Abs to S 100, Abs to TNF-α and Abs to H. The severity of the condition was expressed by the total score and the patients were allocated into one of the six groups: 0–1 – normal (healthy); 2–6 – borderline ill; 7–12 – mildly ill; 13–18 – moderately ill; 19–24 – markedly ill; > 25 – severely ill.

The total score of domains 1 and 2 allows to estimate the intensity of abdominal pain; the total score of domains 3 and 4 allows to estimate the severity of symptoms of early satiety. The dynamics of points of the 5th domain allow us to estimate the intensity of pain, decreasing after bowel emptying, the 6th domain allows us to estimate the intensity of abdominal distention, and the 7th domain allows us to estimate the characteristics of the stool.

### Evaluated parameters

1. The proportion of patients who had a decrease in the total score according to the “7*7” questionnaire by 50% or more after 12 weeks of therapy in the groups with FD, IBS, and FD-IBS overlap.
2. The proportion of patients who switched to a lower severity category of the condition according to the “7*7” questionnaire after 12 weeks of therapy in the groups with FD, IBS, and FD-IBS overlap.
3. The proportion of patients who switched to the “healthy” or “borderline ill” severity categories according to the “7*7” questionnaire after 12 weeks of therapy in the groups with FD, IBS, and FD-IBS overlap.
4. The change in the average score in domains 1–7 according to the “7*7” questionnaire after 12 weeks of therapy in the groups with FD, IBS, and FD-IBS overlap.

### Methods of statistical analysis

No inferential and statistical analyses was used due to absence of groups of comparison to the treatment group. Continuous variables are presented as estimates of mean and categorical variables are presented as a number and percentage of patients in the respective categories. Data from patients with missing values were not included in the analysis.

## Results

### Patient characteristics

A total of 14,362 patients participated in the study. The final efficacy analysis included data from 9254 patients. The data of 5108 patients were not used to assess the effectiveness of the therapy, since 1645 patients had organic gastrointestinal diseases besides the presence of FGID, and 3463 patients had missing data that did not allow for evaluating the dynamics of symptoms. The safety analysis took into account the data of all 14,362 patients.

Among the patients included in the efficacy analysis, 2404 patients were diagnosed with FD, 5909 patients had IBS, and 941 patients had overlapping IBS and FD.

The average age of patients with FD was 33.5 ± 11.2 years, with IBS – 37.8 ± 12.7 years, and with overlapping IBS and FD – 36.3 ± 11.3 years.

Among the participants of the COMFORT program, women prevailed (5898 patients). In the group of patients with FD, there were 1437 (59.78%) women and 967 (40.22%) men, in the group of IBS – 3849 (65.14%) women and 2060 (34.86%) men, and in the group of patients with overlapping IBS and FD, there were 612 (65.04%) women and 329 (34.96%) men.

By severity levels, patients were distributed as follows: 383 patients (4.13%) “borderline ill” (total score 2–6), 2822 patients (30.60%) “mildly ill” (total score 7–12), 3236 patients (34.96%) “moderately ill” (total score 13–18), 1708 patients (18.45%) “markedly ill” (total score 19–24), 1105 patients (11.94%) “severely ill” (total score > 25) (Table 1).

**Table 1:** The distribution of patients categorized by severity of symptoms according to the “7*7” questionnaire before treatment with the combination of RAF of Abs to S 100, Abs to TNF-α and Abs to H

Severity category	Before treatment, n (%)
Healthy	0
Borderline ill	383 (4.13%)
Mildly ill	2822 (30.60%)
Moderately ill	3236 (34.96%)
Markedly ill	1708 (18.45%)
Severely ill	1105 (11.94%)

The distribution of patients by categories of severity and by various types of diseases is presented in Table 2.

**Table 2:** The categorization of severity among patients with FD, IBS, and FD-IBS overlap before treatment with the combination of RAF of Abs to S 100, Abs to TNF-α and Abs to H

Group		Severity category					
		Healthy	Borderline ill	Mildly ill	Moderately ill	Markedly ill	Severely ill
FD *N* = 2404	Before therapy n (%)	0 (0.0)	161 (6.69)	778 (32.36)	740 (30.78)	431 (17.92)	294 (12.22)
IBS *N* = 5909	Before therapy n (%)	0 (0,0)	214 (3.62)	1911 (32.34)	2113 (35.75)	1036 (17.53)	635 (10.74)
FD-IBS overlap *N* = 941	Before therapy n (%)	0 (0.0)	8 (0.85)	133 (14.13)	383 (40.70)	241(25.61)	176(18.70)

### Efficacy evaluation

According to the “7*7” questionnaire after 12 weeks of treatment with the combination of RAF of Abs to S 100, Abs to TNF-α and Abs to H, a decrease in the number of points by 50% was observed in 80.45% of patients with FD, 79.02% of patients with IBS, and in 83% of patients in the group with FD-IBS overlap (Table 3).

**Table 3:** The absolute number of patients with FD, IBS, and FD-IBS overlap, in which there was a decrease in the total score according to the questionnaire “7*7” by 50% or more after 12 weeks of therapy with the combination of RAF of Abs to S 100, Abs to TNF-α and Abs to H

Group	The total number of patients in the group	The number of patients in the group who had a decrease in the total score by 50% or more, n (%)
FD	2404	1934 (80.45%)
IBS	5909	4669 (79.02%)
FD-IBS overlap	941	781 (83%)

A decrease in the severity category of the condition at the end of therapy was noted in 93.34% of cases in patients with FD, in 93.81% of cases in patients with IBS, and in 96.17% of cases in patients with overlapping IBS and FD.

**Fig. 1:**
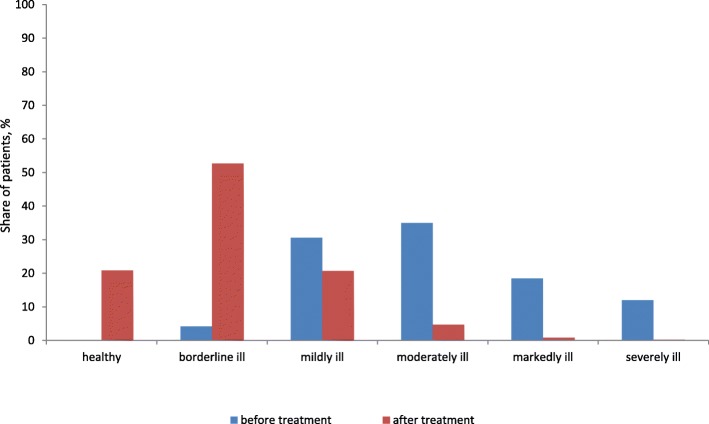
The proportion of patients with FD, IBS, and FD-IBS overlap, categorized by severity of symptoms according to the “7*7” questionnaire, before and after 12 weeks of treatment with the combination of RAF of Abs to S 100, Abs to TNF-α and Abs to H

After 12 weeks of treatment with the combination of RAF of Abs to S 100, Abs to TNF-α and Abs to H, the distribution of patients according to the severity categories was as follows: 1930 patients (20.85%) comprised the group “healthy”, 4871 patients (52.63%) –“borderline ill”, 1915 patients (20.69%) – “mildly ill”, 435 patients (4.70%) – “moderately ill”, 78 patients (0.84%) – “markedly ill”, and 25 patients (0.27%) – “severely ill” (Table 4, Fig. 1).

**Table 4:** The distribution of patients categorized by severity of symptoms according to the “7*7” questionnaire before and after 12 weeks of therapy with the combination of RAF of Abs to S 100, Abs to TNF-α and Abs to H

Severity category	Before treatment, n (%)	After 12 weeks of therapy, n (%)
Healthy	0	1930 (20.85%)
Borderline ill	383 (4.13%)	4871 (52.63%)
Mildly ill	2822 (30.60%)	1915 (20.69%)
Moderately ill	3236 (34.96%)	435 (4.70%)
Markedly ill	1708 (18.45%)	78 (0.84%)
Severely ill	1105 (11.94%)	25 (0.27%)

The distribution of patients according to severity categories in the groups with FD, IBS, and FDIBS overlap after therapy with the combination of RAF of Abs to S 100, Abs to TNF-α and Abs to H is presented in Table 5.

**Table 5:** The categorization of severity among patients with FD, IBS, and FD-IBS overlap before and after therapy with the combination of RAF of Abs to S 100, Abs to TNF-α and Abs to H

Group		Severity category					
		Healthy	Borderline ill	Mildly ill	Moderately ill	Markedly ill	Severely ill
FD N = 2404	Before therapy n (%)	0 (0.0)	161 (6.69)	778 (32.36)	740 (30.78)	431 (17.92)	294 (12.22)
	After 12 weeks of therapy, n (%)	537(22.33)	1339 (55.69)	406 (16.88)	99 (4.11%)	17 (0.70)	6 (0.24)
IBS N=5909	Before therapy n (%)	0 (0.0)	214 (3.62)	1911 (32.34)	2113 (35.75)	1036 (17.53)	635 (10.74)
	After 12 weeks of therapy, n (%)	1237 (20.93)	3044 (51.51)	1304 (22.06)	267 (4.51)	43 (0.72)	14 (0.23)
FD-IBS overlap N = 941	Before therapy n (%)	0 (0.0)	8 (0.85)	133 (14.13)	383 (40.70)	241(25.61)	176(18.70)
	After 12 weeks of therapy, n (%)	156 (16.57)	488 (51.85)	205 (21.78)	69 (7.33)	18 (1.91)	5(0.53)

In 159 (6.61%) patients with FD, there was no change in the severity of symptoms; in 1 patient (0.04%), there was a worsening of the state of health. The degree of severity of symptoms remained unchanged after therapy with the combination of RAF of Abs to S 100, Abs to TNF-α and Abs to H in 365 patients (6.18%) with IBS; in 1 patient (0.02%), a switch to a more “severe” group was observed. Symptom intensity did not change in 36 patients (3.83%) with FD-IBS overlap; there were no patients who switched to a more “severe” category in this group.

**Fig. 2:**
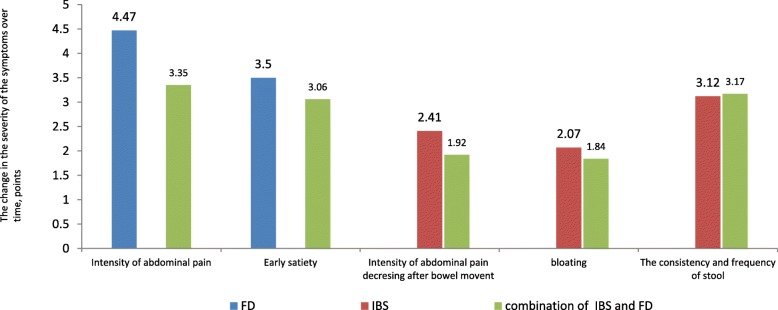
The change in the number of points characterizing the severity of symptoms

In 2127 (88.48%) patients with FD, there was a change in the score in domains 1 and 2, which characterize pain and a burning sensation in the upper middle part of the abdomen. The average score in domains 1 and 2 decreased by 4.47. In 2115 patients with FD (87.97%), the average score in domains 3 and 4, which characterizes the syndrome of early satiety, also decreased by 3.5 (Table 6, Fig. 2).

**Table 6:** The average score characterizing the severity of symptoms and their dynamics in domains 1–7 in patients with FD, IBS, and FD-IBS overlap

Domain	Symptom	Patient groups								
		FD		Severity dynamics	IBS		Severity dynamics	FD-IBS overlap		Severity dynamics
		Before treatment, n (%)	After treatment		Before treatment, n (%)	After treatment		Before treatment, n (%)	After treatment	
1 + 2	Intensity of abdominal pain	6.16	1.69	4.47	–	–	–	4.73	1.38	3.35
3 + 4	Early satiety	4.67	1.52	1.92	–	–	–	10.08	3.14	3.06
5	Intensity of abdominal pain after bowel movement	–	–	–	3.37	0.96	2.41	2.76	0.84	1.92
6	Bloating	–	–	–	3.11	1.04	2.07	2.77	0.93	1.84
7	The consistency and the frequency of stool	–	–	–	4.78	1.66	3.12	4.55	1.37	3.17

In 5017 (84.9%) patients with IBS, there was a decrease in the score in domain 5, which characterizes abdominal pain that decreases after a bowel movement. The decrease in the average score in this group was 2.41. Also in the majority of patients in the IBS group (87.31%), there was a decrease in the score in domain 6, indicating a decrease in complaints of bloating. The average score in domain 6 decreased by 2.07. After 12 weeks of treatment with the combination of RAF of Abs to S 100, Abs to TNF-α and Abs to H, 5123 (86.70%) patients reported a decrease in the number of complaints about the consistency and frequency of the stool. The average score in domain 7 decreased by 3.12 (Table 6, Fig. 2).

In patients with FD-IBS overlap, a decrease in the average score in each of the 7 domains was found. A decrease in pain and burning sensation was noted in 774 (82.25%) patients with FD-IBS overlap: the average score in domains 1 and 2 decreased by 3.35. A decrease in the score in domains 3 and 4 (characterizing the syndrome of early satiety) was found in 783 (83.21%) patients. In this category of patients, the average score decreased by 3.06. 725 (77.05%) patients noted a decrease in the severity of pain after bowel movement. The average score in domain 5 decreased by 1.92. After 12 weeks of treatment with the combination of RAF of Abs to S 100, Abs to TNF-α and Abs to H, 772 (82.04%) patients had a decrease in the score in domain 6, indicating a reduction in number of complaints of bloating. In this group, the average score decreased by 1.84 points. For 830 (88.20%) patients, there was a decrease in scores in domain 7, which characterizes a change in the consistency and frequency of stools. The decrease in the average score was 3.17 (Table 6, Fig. 2).

### Safety evaluation

A total of 94 adverse events (AEs) were recorded (Table 7) in 80 patients (0.65%); or less than 1 case per 100 patients [25]. Most adverse events (55 cases, or 58.51% of all AEs) were associated with dysfunction of the digestive organs. Nausea was recorded most frequently, 22 cases of AE, even though overall this type of AE is “infrequent” (0.15%—i.e., less than 1 case per 100 patients), according to doctors, due to the nature of the course of FGID.

**Table 7:** Recorded Adverse Events

System organ class MedDRA	Disease	Number of AE	% of all patients	% of all AE
	Total AE	94	0.65	100.00
	N of patients who had at least 1 AE	80	0.55	
Skin and subcutaneous tissue disorders	Erythema	1	0.007	
	Pruritus	6	0.04	
	Rash	1	0.007	
	Urticaria	2	0.01	
	N of AE in this group	10	0.06	10.63
	N of patients who had at least 1 AE in this group	8	0.05	
Musculoskeletal and connective tissue disorders	Myalgia	2	0.0139	
	N of AE in this group	2	0.0139	2.12
	N of patients who had at least 1 AE in this group	2	0.0139	
Nervous system disorders	Dizziness	3	0.02	
	Dysgeusia	2	0.01	
	Head discomfort	1	0.007	
	Headache	11	0.07	
	N of AE in this group	17	0.11	18.09
	N of patients who had at least 1 AE in this group	15	0.10	
Digestive system disorders	Abdominal distension	2	0.01	
	Abdominal pain	14	0.09	
	Abdominal pain upper	1	0.007	
	Anal pruritus	1	0.007	
	Constipation	4	0.02	
	Diarrhoea	2	0.01	
	Dyschezia	1	0.007	
	Dyspepsia	1	0.007	
	Epigastric discomfort	3	0.0209	
	Flatulence	1	0.007	
	Nausea	22	0.15	
	Tongue discomfort	3	0.02	
	N of AE in this group	55	0.38	58.51
	N of patients who had at least 1 AE in this group	51	0.35	
Cardiac disorders	Palpitations	1	0.007	
	N of AE in this group	1	0.007	1.06
	N of patients who had at least 1 AE in this group	1	0.007	
General disorders and administration site conditions	Asthenia	2	0.01	
	Drug ineffective	1	0.007	
	Feeling jittery	1	0.007	
	N of AE in this group	4	0.02	4.25
	N of patients who had at least 1 AE in this group	3	0.02	
Psychiatric disorders	Agitation	2	0.01	
	Sleep disorder	1	0.007	
	N of AE in this group	3	0.02	3.19
	N of patients who had at least 1 AE in this group	2	0.01	
Metabolism and nutrition disorders	Decreased appetite	1	0.007	
	Increased appetite	1	0.007	
	N of AE in this group	2	0.01	2.12
	N of patients who had at least 1 AE in this group	2	0.01	

Less commonly recorded AEs involved the nervous system: 17 cases or 18.09% of all identified AEs, and were also “infrequent” (0.11%).

AEs associated with skin diseases and subcutaneous fat were uncommon: 10 cases or 10.63% of all identified AEs, which were “rare” (0.06%, less than 1 case per 1000 patients).

Also recorded were general disorders (4 AEs, or 4.25% of all identified AEs), nutritional and metabolic disorders (2 AEs, or 2.12% of all identified AEs), mental disorders and behavioral disorders (3 AEs, or 3.19% of all identified AEs)—all classified as “rare”.

Two AEs associated with the musculoskeletal system and connective tissue (2.12% of all identified AEs) and 1 AE associated with the circulatory system (1.06% of all identified AEs) were recorded as well.

During the study period, no major AEs were identified. Also, no patients stopped taking the combination of RAF of Abs to S 100, Abs to TNF-α and Abs to H during the observation period.

## Discussion

The COMFORT Observation Program has been completed in Russia with the participation of 14,362 patients. This program evaluated the efficacy and safety of the use of the combination of RAF of Abs to S 100, Abs to TNF-α and Abs to H in patients with FD, IBS, and FD-IBS overlap.

The distribution of patients with the FGID by gender in the COMFORT program corresponded to the data of population studies in Europe and North America [26, 27].

According to the literature, the majority of patients with FGID have abdominal symptoms of mild to moderate severity [28]. Similar trend was also observed in the present study: the majority of patients at the stage of inclusion described the severity of symptoms as “moderately ill”.

The obtained results demonstrate that the combination of RAF of Abs to S 100, Abs to TNF-α and Abs to H has a pronounced therapeutic effect, reducing the intensity of symptoms of FGID by more than half in 80% of patients, which is consistent with previously obtained data in a multicenter, double-blind, placebo-controlled, randomized clinical trial of the efficacy and safety of using the combination of RAF of Abs to S 100, Abs to TNF-α and Abs to H for treating patients with IBS [23]. Along with a decrease in the intensity of abdominal pain, there was a normalization of the frequency and consistency of the stool, indicating a restoration of the motor-evacuation function of the GIT [23].

The previous study has also shown that the combination of RAF of Abs to S 100, Abs to TNF-α and Abs to H is effective in the treatment of patients with FD-IBS overlap: 12 weeks of therapy reduced the incidence of abdominal distention and nausea by 1.5 and 3 times compared with placebo [23].

According to Chen, the presence of coexisting FGID syndromes worsens their prognosis [29]. Long-term, prospective observation of patients with FD-IBS overlap showed that only 12% of them were able to achieve stable remission [30]. Therapy with the combination of RAF of Abs to S 100, Abs to TNF-α and Abs to H led to a greater increase in the number of patients in the “healthy” and “borderline ill” categories. The proportion of patients classified as “healthy” was 22.33% (537) in FD group; 20.93% (1237) of in IBS group, and 16.57% (156) in FD-IBS overlap group. A “borderline ill” cohort consisted of 1339 patients (55.69%) with FD, 3044 patients (51.51%) with IBS, and 488 (51.85%) patients with FD-IBS overlap (Table 5).

Thus, the proportion of patients with no clinical manifestations of FGID was 77% in FD group, 71% in IBS group, and 68% in FD-IBS overlap group. Our findings contrast with the results of a systematic review of 22 studies evaluating the effectiveness of 12 antispasmodics in relieving symptoms of IBS in 1778 patients which revealed that 39% of patients have persistent symptoms after therapy [31].

The observational program COMFORT showed a positive effect of the combination of RAF of Abs to S 100, Abs to TNF-α and Abs to H in the majority of patients with FD-IBS overlap: in 83% of cases, there was a decrease in the total score of the “7*7” questionnaire by 50% or more. These results confirm the previously obtained data that treatment with the combination of RAF of Abs to S 100, Abs to TNF-α and Abs to H has a corrective effect on the manifestation of visceral sensitivity and nociceptive dysfunction [23].

Used as a tool for assessing the severity of symptoms of FD, the “7*7” questionnaire is convenient for the doctors and does not take much time from the patients [24]. According to the experience of practicing physicians, a detailed interview of a patient with FGID cannot take less than 45–60 min [32]. The “7*7” questionnaire used in this observational program is able to significantly minimize the time spent by a doctor when there is insufficient time allotted for the examination of the patient.

Similar international scales are often difficult to understand, need a long time to fill out the questionnaire, and are cluttered with terminology [33, 34].

The “7*7” questionnaire meets the requirements of the European Medical Agency, which recommends separately monitored stool frequency, bowel movement consistency, the severity of abdominal pain, and abdominal distention [35].

### Limitations

There are limitations to our study that should be considered. Firstly, the observational nature of the program did not suggest the presence of a comparison group. However, the value of this study is the maximum proximity to actual clinical practice and the possibility of obtaining additional data on the effectiveness of the drug in various FGID, including when they are combined.

Secondly, in the COMFORT program, patients with IBS were not subdivided into subtypes of IBS.

Inability to include the data of 5108 patients can be also considered as a limitation of the current study. At the same time, we should emphasize that some doctors might have experienced some difficulties with the filling of the various forms, thus the information on 3463 patients was missing. As previously mentioned, the data of 1645 patients with concomitant organic diseases of the GIT were not taken into account in the analysis of efficiency, since the overlap of these diagnoses can occur in case of their incorrect differential diagnosis. Furthermore, in accordance to Rome IV criteria these cases might be considered as secondary dyspepsia, which goes against the inclusion criteria. Another limitation of our study is related to the fact that the “7*7”questionnaire is a validated scale used only in Russia and is lacking international acceptance. Based on the accumulated experience, the ‘7 × 7’ questionnaire can be recommended for use in clinical practice. It has been validated as a convenient, sensitive and reliable tool for assessing the severity of symptoms and their dynamics in treating patients with FGID, as well as for evaluating not only the improvement of the condition as the result of the treatment, but also the absence of changes or deterioration of the condition.

## Conclusions

The results of the COMFORT observational program demonstrated the effectiveness of the combination of RAF of Abs to S 100, Abs to TNF-α and Abs to H in treating patients with FD, IBS, and overlapping IBS and FD. In the absence of clear recommendations on overlap syndrome pharmacotherapy, an important conclusion of the study was the evidence of effective treatment of such patients.

The combination of RAF of Abs to S 100, Abs to TNF-α and Abs to H demonstrated good tolerability and the absence of negative effects on the patient’s condition, which is important for long-term therapy of FGID.

## Data Availability

The conducted postmarketing surveillance study included data of 14362 outpatient’s medical records. It is technically difficult to transfer such a vast amount of data. However, we are ready to give access to the data at the request of the reviewer after the evaluation of this article. In such case, we would kindly ask to provide us a non-disclosure agreement.

## Abbreviations

Abs: Antibodies
AEs: Adverse events
FD: Functional dyspepsia
FGID: Functional gastrointestinal disorders
GIT: Gastrointestinal tract
IBS: Irritable bowel syndrome
IL: Interleukin
LLC “NPF “MATERIA MEDICA HOLDING”: Limited Liability Company Research and Production Company Materia Medica Holding
RAF of Abs to H: RAF of Abs to histamine
RAF of Abs to S-100: RAF of antibodies to S 100 protein
RAF of Abs to TNF-α: RAF of antibodies to tumor necrosis factor-α
RAF: Released-active form
TNF-α: Tumor necrosis factor-α
